# Differences in Chemical Composition of Soil Organic Carbon Resulting From Long-Term Fertilization Strategies

**DOI:** 10.1371/journal.pone.0124359

**Published:** 2015-04-17

**Authors:** Zengqiang Li, Bingzi Zhao, Qingyun Wang, Xiaoyan Cao, Jiabao Zhang

**Affiliations:** 1 State Key Laboratory of Soil and Sustainable Agriculture, Institute of Soil Science, Chinese Academy of Sciences, Nanjing, China; 2 Graduate School of the Chinese Academy of Science, Beijing, China; 3 Department of Chemistry, Brandeis University, Waltham, Massachusetts, United States of America; Old Dominion Univ., UNITED STATES

## Abstract

Chemical composition of soil organic carbon (SOC) is central to soil fertility. We hypothesize that change in SOC content resulting from various long-term fertilization strategies accompanies the shift in SOC chemical structure. This study examined the effect of fertilization strategies along with the time of fertilizer application on the SOC composition by ^13^C nuclear magnetic resonance (NMR) spectroscopy. The soils (Aquic Inceptisol) subjected to seven fertilizer treatments were collected in 1989, 1999 and 2009, representing 0, 10 and 20 years of fertilization, respectively. The seven fertilizer treatments were (1–3) balanced fertilization with application of nitrogen (N), phosphorus (P) and potassium (K) including organic compost (OM), half organic compost plus half chemical fertilizer (1/2OM), and pure chemical NPK fertilizer (NPK); (4–6) unbalanced chemical fertilization without application of one of the major elements including NP fertilizer (NP), PK fertilizer (PK), and NK fertilizer (NK); and (7) an unamended control (CK). The SOC content in the balanced fertilization treatments were 2.3–52.6% and 9.4–64.6% higher than in the unbalanced fertilization/CK treatments in 1999 and 2009, respectively, indicating significant differences in SOC content with time of fertilizer application between the two treatment groups. There was a significantly greater proportion of O-alkyl C and a lower proportion of aromatic C in the balanced fertilization than in unbalanced fertilization/CK treatments in 1999, but not in 2009, because their proportions in the former treatments approached the latter in 2009. Principal component analysis further showed that the C functional groups from various fertilization strategies tended to become compositionally similar with time. The results suggest that a shift in SOC chemical composition may be firstly dominated by fertilization strategies, followed by fertilization duration.

## Introduction

Soil fertility is related to the chemical composition of soil organic carbon (SOC) [1–5]. In a pine plantation, Mathers and Xu [1] showed a significantly positive correlation of potentially mineralizable nitrogen (N) with O-alkyl C proportion and a negative correlation between available phosphorus (P) content and the aromatic C/O-alkyl C ratio. The proportions of alkyl C to O-alkyl C in forest soils have been found to be linearly related to cumulative N mineralization [2]. Among SOC fractions, hot-water extractable organic matter and particulate organic matter have been found to be enriched in the O-alkyl C component [3, 4]. Ng et al. [5] reported that abundances of alkyl C, O-aryl C, aryl C and carbonyl C have been used to explain most of the variations (>50%) observed in soil microbial community composition and activity. Among these, carbonyl-C content (representing relatively labile C forms) strongly affected the microbial activity.

Long-term fertilization strategies have been shown to significantly influence the SOC chemical composition. Relative to mineral fertilizer treatments, organic manure application decreased aromatic C abundance and alkyl C/O-alkyl C ratio of SOC after 4 or 21 years of continuous fertilization [6, 7]. Soil particulate organic matter contained relatively less O-alkyl C, but more aromatic C, under 18-years of chemical fertilizer plus pig manure treatment compared to unfertilized treatment [8]. Moreover, 25 years of continuously unbalanced fertilization with N and P fertilizers led to decreased abundances of O-alkyl C likely due to microbial decomposition and increased abundances of alkyl C in the humin fraction of soil [9]. However, Yan et al. [10] observed nearly no differences in SOC chemical composition in paddy soil or upland soil between 29- or 24-year balanced and unbalanced chemical fertilization. Clearly, ambiguity exists in the literature, which hinders making any estimate of the effect of various fertilization strategies on SOC chemical composition.

The North China Plain (NCP) is one of the major agricultural production areas in China. Low content of SOC (usually <10 g kg^–1^) mainly limits crop production in the plain [11]. Therefore, application of various organic and inorganic fertilizers is necessary to sustain crop production and soil quality. When farmers cannot afford balanced fertilizers, only N and/or P fertilizers are applied whereas the need for potassium (K) is either underestimated or completely ignored [12]. Consequently, unbalanced fertilization without one of the major elements (N, P and K) is still widespread in the NCP.

Previous studies based on long-term field fertilization experiments initiated in 1989 in the NCP revealed that long-term continuous application of organic compost generally led to greater SOC accumulation, amounts of microbial biomass, microbial activities, and stability of function than chemical fertilization alone, as well as promoting growth of indigenous bacteria in soil [11–13]. In addition, microbes showed more efficient metabolism as indicated by heat dissipation per cell unit under balanced than unbalanced fertilization [14]. These observations have been attributed largely to the corresponding changes in SOC content. We hypothesize that a shift in SOC chemical structure may accompany the change in the SOC content under long-term fertilization strategies, which has not previously been emphasized or addressed before in the NCP.

Utilizing the aforementioned long-term fertilization experiments located in the NCP, the primary objective of this study is to evaluate the effects of 10 and 20 years of various continuous fertilization strategies, including organic compost application and balanced and unbalanced chemical fertilization, on SOC chemical composition in the NCP using ^13^C cross polarization magic angle spinning nuclear magnetic resonance (^13^C CPMAS NMR) spectroscopy. The main assumption in this study is that the SOC content and degradation pathways change with fertilization treatments and time of fertilizer application. The specific objectives are to: (1) determine SOC composition changes caused by different fertilization strategies and (2) investigate whether such changes are time-related/dependent.

## Materials and Methods

### Study site and soil sampling

The long-term experimental field site is located in the Fengqiu Agroecological Experimental Station of the Chinese Academy of Sciences in Pandian, Fengqiu County, Henan Province of China (114°24′ E, 35°00′ N). The mean annual air temperature is 14.5°C, with the lowest average temperature of—1.0°C occurring in January and the highest temperature of 27.2°C in July. The average annual precipitation is 615 mm, 60–90% of which is during May–October. The soil was derived from alluvial sediments of the Yellow River, and is classified as an Aquic Inceptisol, containing 52% sand, 33% silt and 15% clay. Before the experiment started in 1989, the soil contained 4.48 g kg^-1^ organic C, 0.43 g kg^-1^ total N, 0.50 g kg^-1^ total P, 18.6 g kg^-1^ total K, 9.51 mg kg^-1^ available N, 1.93 mg kg^-1^ available P, and 78.8 mg kg^-1^ available K. The field had been under winter wheat (*Triticum aestivum* L.) and summer maize (*Zea mays* L.)—two crops in a one-year rotation. Winter wheat is grown from October to May and summer maize from June to September each year.

The experiments were initiated in October 1989, when winter wheat was sown, to investigate the effects of application of organic compost and balanced and unbalanced chemical fertilizers without one of the major elements on crop yield and soil fertility. Nutrient application amounts were as follows: N = 150 kg ha^-1^, P = 32.7 kg ha^-1^, and K = 124.5 kg ha^-1^ for winter wheat, and N = 150 kg ha^-1^, P = 26.2 kg ha^-1^, and K = 124.5 kg ha^-1^ for summer maize. Briefly, the experiments contained seven fertilizer treatments: (1) OM: all required N was provided by application of organic compost, prepared by mixing wheat straw, rapeseed cake and cottonseed cake in a ratio of 100:40:45 and fermenting the mixture for two months. The compost contained 422 g kg^-1^ organic C, 54.4 g kg^-1^ total N, 8.1 g kg^-1^ total P, and 19.5 g kg^-1^ total K, with a C:N ratio of 7.8. The amounts of P and K contained in the compost were generally less than the required application amounts, and were supplemented with chemical fertilizers to achieve the required P and K contents, (2) 1/2OM: half of the required N was provided by the organic compost and the other half by the chemical fertilizers, with P and K supplemented to the required amounts, (3) NPK: balanced chemical fertilization with N, P and K, (4) NP: N and P were applied without K, (5) PK: P and K were applied without N, (6) NK: N and K were applied without P, and (7) CK: no fertilizers were applied. A completely randomized block design with seven fertilizer treatments and four replications was used. More details are described elsewhere [12].

Soil samples from the plow layer (0–20 cm) were collected once a year after maize was harvested and some basic soil properties were determined for seven fertilizer treatments [15]. The remaining soils were air dried and stored., The soil samples used in the present study were collected in 1989 (i.e. the year when the experiments were initiated), 1999 and 2009, representing 0, 10 and 20 years of various continuous fertilization, respectively. SOC and ^13^C NMR analyses were done in triplicate.

### Soil organic carbon analysis and solid-state ^13^C NMR spectroscopy

Soil organic carbon content was analyzed using a potassium dichromate method [16].

Soil samples for solid-state ^13^C NMR analysis were repeatedly treated with 2% hydrofluoric acid (HF) solution to concentrate the organic matter content and to remove paramagnetic minerals [17]. Briefly, 5 g of air-dried soil samples (<0.2 mm) were added into 100 mL polyethylene tubes. After the addition of 50 mL of 2% HF solution, the tubes were closed and shaken for 1 h, followed by centrifugation for 20 min at 2000 rpm at room temperature. The supernatant was then removed and discarded. The residue was again submitted to HF treatment as described above. Finally, samples were repeatedly treated eight times with shaking times of 5 × 1 h, 2 × 16 h and 1 × 64 h. After the final extraction, the residue was washed for three times using distilled water, followed by drying at 75°C, and sieving through 0.2 mm for ^13^C NMR analysis.

The chemical composition of SOC was characterized using solid-state ^13^C NMR spectroscopy. Cross polarization with magic angle spinning (CPMAS) was combined with total suppression of spinning sidebands (TOSS) to enhance sensitivity and remove overlap of spinning sidebands and isotropic resonances [18]. Previous work had established that spinning sidebands made a contribution to the CPMAS spectra in the aromatic region, so we decided to run TOSS on all the samples. The solid-state ^13^C NMR spectra were obtained on a Bruker AVANCE III 400 (Bruker, Fallandenat, Switzerland) operating at a ^13^C resonance frequency of 100.62 MHz. Samples in a zirconia rotor were spun at 6 kHz within a 7 mm MAS probe (Bruker). An acquisition time of 10.3 ms, and a contact time of 1 ms with a spectral width of 100 kHz were used. The recycle time was set at 0.5 s. The number of required scans was about 90,000. In the preliminary work, we could not find the background signal of the empty rotor using the CP/TOSS technique. The ^13^C chemical shifts were externally referenced to adamantine at 38.48 ppm. The relative contents of different C functional groups were obtained by integrating signal intensities within various chemical shift regions, and expressed as percentages of the total area (0–210 ppm).

According to previous NMR studies of SOC [1, 19–22], the ^13^C NMR spectra of soil samples are generally assigned to the following eight dominant C forms: (1) alkyl C (0–45 ppm), (2) *N*-alkyl/methoxyl C (45–60 ppm), (3) carbohydrate C (60–90 ppm), (4) di-O-alkyl C (90–110ppm), (5) aryl C (110–145 ppm), (6) O-aryl C (145–160 ppm), (7) carboxyl/amide C (160–190 ppm), and (8) ketone/aldehyde C (190–210 ppm), in which (2), (3), and (4) can be combined as O-alkyl C (45–110 ppm), (5) and (6) as aromatic C (110–160 ppm), and (7) and (8) as carbonyl C (160–210 ppm).

### Statistical analysis

One-way analysis of variance (ANOVA) was used to test the differences in SOC content, and relative contents of various functional groups among the fertilization treatments with increasing time of continuous fertilizer application. Prior to each ANOVA, normality was checked using the Shapiro–Wilk test. Homogeneity of variance was verified using Levene’s test. The Tukey post hoc test was used following ANOVAs for multiple comparisons, with statistical difference level set at 5%. The abundances of eight C types in 1999 and 2009 soil samples were subjected to principal component analysis (PCA), separately, using a covariance matrix, to differentiate the effects of various fertilization treatments. All statistical analyses were performed using SPSS version 17.0.

## Results

### Soil organic carbon (SOC)

**Fig 1:**
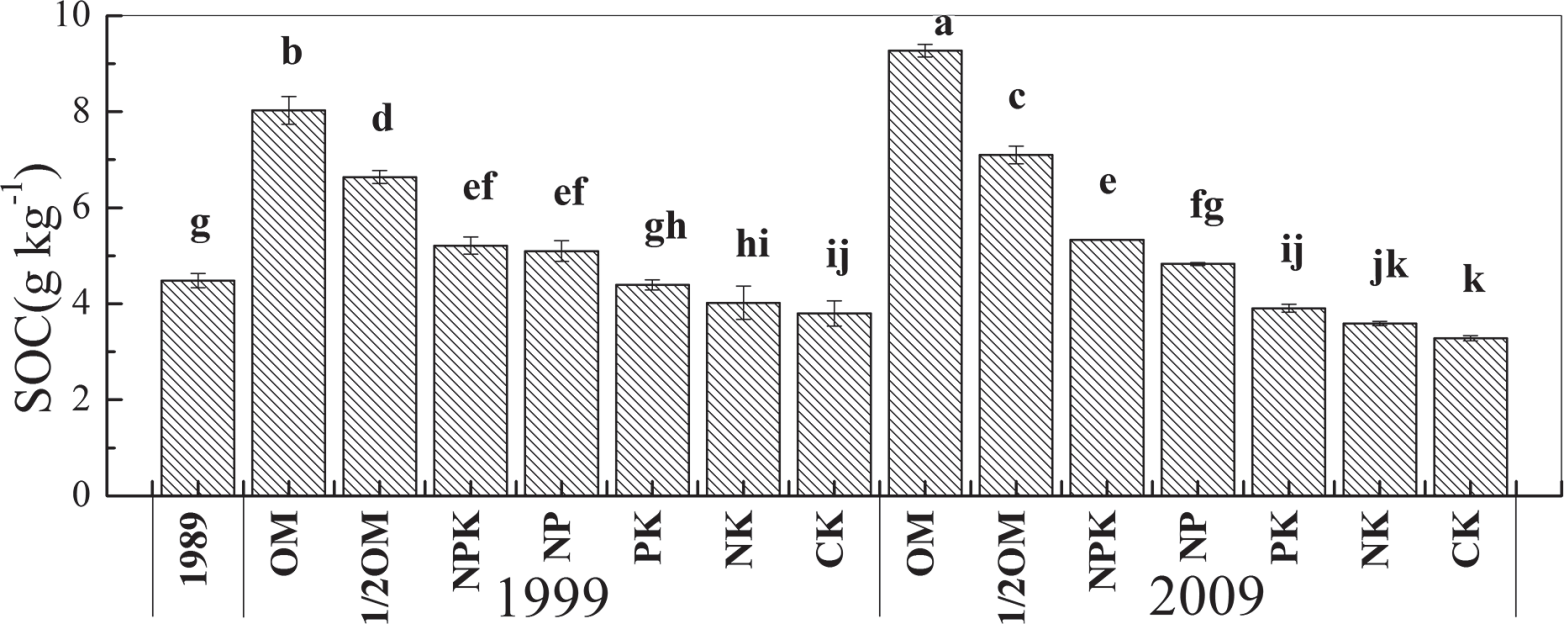
Soil organic carbon (SOC) content as affected by fertilization treatment and time. Treatments followed by different letters are significantly different at *p* < 0.05 (Tukey test, n = 3).

The average SOC content among different fertilization treatments in 1999 decreased in the order of OM > 1/2OM > NPK, NP > PK > NK, CK, and in 2009 the order of OM > 1/2OM > NPK > NP > PK > NK, CK (Fig 1). Generally, the SOC contents from the balanced fertilization (i.e. OM, 1/2OM and NPK) treatments were 2.3–52.6% and 9.4–64.6% higher than from other treatments in 1999 and 2009, respectively.

Compared to the SOC content in 1989 when the experiments started, the SOC content in 1999 and 2009 increased by 16.3–79.0% and 19.0–107%, respectively, in the balanced fertilization; and correspondingly increased by 13.6 and 7.8% in the NP and decreased by 1.8–15.2% and 12.9–26.8% in the PK, NK and CK treatments (Fig 1). Therefore, the balanced fertilization treatments significantly increased the SOC content, and this effect became more prominent with time. In contrast, the unbalanced fertilization with PK and NK treatments and CK consistently decreased the SOC content over time. The SOC content in NP treatment increased initially in 1999 (5.09 g kg^–1^) relative to 1989 (4.48 g kg^–1^), but did not continue to increase in 2009 (4.83 g kg^–1^) (Fig 1). These results indicated that the balanced fertilization treatments led to accumulation of significantly more SOC than the unbalanced and CK treatments following the first 10 years of continuous fertilization, and this accumulation effect was more pronounced after the second 10 years continuous fertilization.

### ^13^C NMR spectroscopy

**Fig 2:**
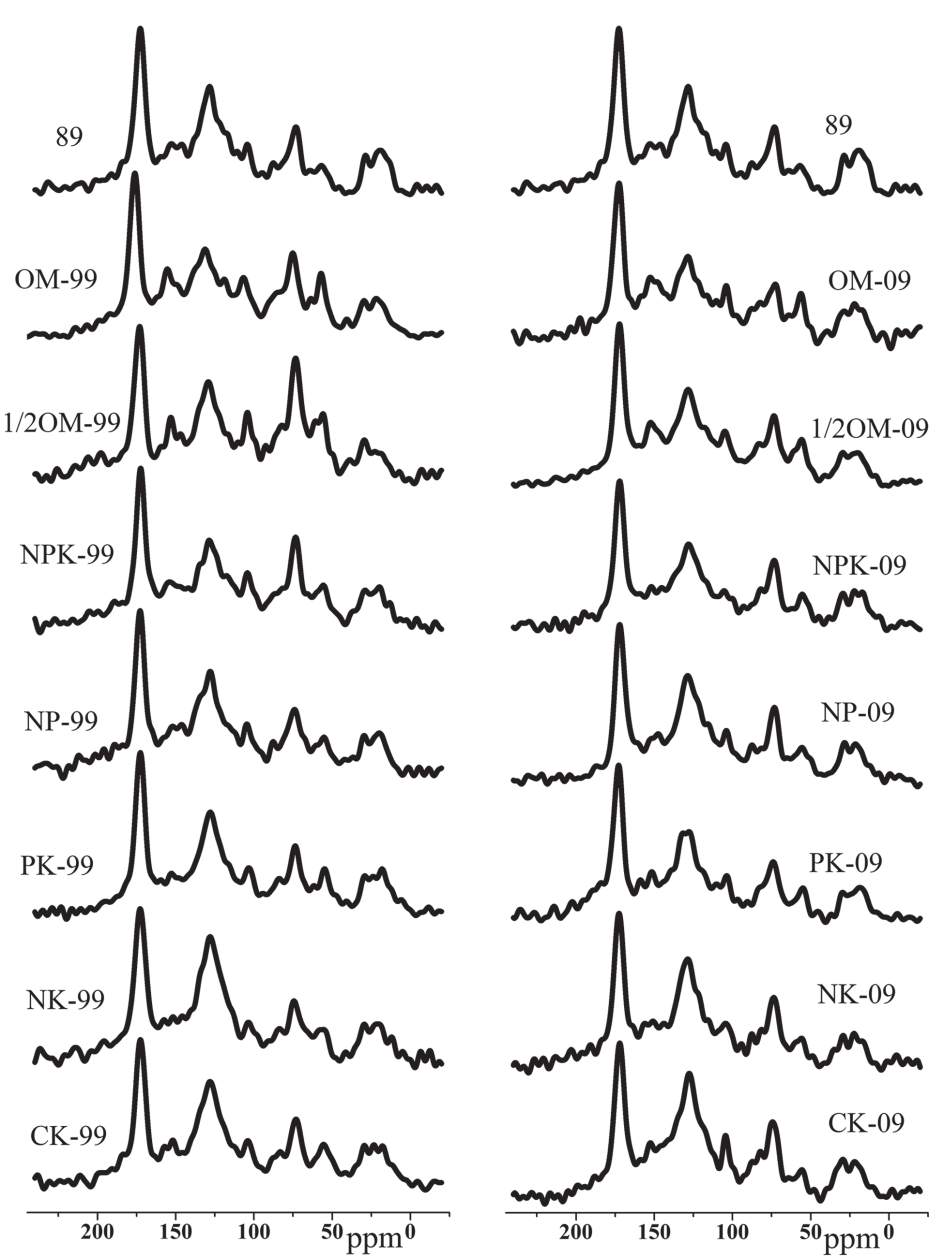
Solid-state CPMAS ^13^C NMR spectra of the soils collected in 1989, 1999 and 2009, separately. 89 = soils collected in 1989, 99 = soils collected in 1999, 09 = soils collected in 2009.

The ^13^C-NMR spectra of soils in 1989 and for seven different treatments during 1999–2009 are displayed in Fig 2. The major NMR bands and their chemical shifts seemed to be consistent among soils with different fertilization strategies or time, due to the general similarity of the constituent functional groups in soil organic matter. The alkyl C (0–45 ppm) region showed signals from CH_2_ groups in long-chain polymethylene structures (e.g. fatty acids, waxes and biopolyesters) and terminal methyl groups from both alkyl compounds and acetyl substituents in plant hemicellulose [23]. In the O-alkyl region (45–110 ppm), the peak at 56 ppm assigned to the methoxyl C/*N*-alkyl groups, are usually derived from guaiacyl and syringyl lignin derivatives, and/or C-N bonds in amino acids, respectively [24]. The signal at 74 ppm represented the overlapping resonances of C-2, C-3 and C-5 carbons in the pyranoside structure of cellulose and hemicelluloses of polysaccharides [24]. The peak at 104 ppm was associated with the anomeric C-1 carbon of cellulose and hemicelluloses present in fresh plant material [25]. The broad band around 130 ppm was attributed to the presence of lignin or partially degraded lignin structures, and condensed aromatic and olefinic C [26]. The small peak in the O-aryl C region (145–160 ppm) was more evident in spectra of some soil samples (OM-99, OM-09, 1/2OM-99 and 1/2OM-09) than others, further confirming the presence of lignin components [26]. Finally, the sharp signal at 172 ppm indicated that carboxyl groups (in aliphatic acids of plant and microbial origins) and/or amide groups (in amino acid moieties) were present in great abundance [20].

Table 1 shows that aromatic C accounted for the most of the total C (33.3–40.3%), followed by O-alkyl C (23.2–33.9%), carbonyl C (23.1–29.1%) and alkyl C (7.8–11.9%). In 1999, the balanced N, P and K fertilization treatments (i.e. OM, 1/2OM and NPK) generally resulted in larger proportions of *N*-alkyl/methoxyl C, carbohydrate C and di-O-alkyl C, i.e. O-alkyl C, than the unbalanced fertilization and CK treatments (Table 1). In contrast, the aryl C, generally accounting for >73% of aromatic C, was significantly less abundant in the balanced than in unbalanced and CK treatments. These trends, however, were not noted among balanced and unbalanced fertilization and CK treatments in 2009 (Table 1).

**Table 1:** Composition of C functional groups (%) estimated from relative integration values of ^13^C CPMAS NMR spectra of soil samples.

	Alkyl C (0–45ppm)	O-alkyl C (45–110 ppm)				Aromatic C (110–160 ppm)			Carbonyl C (160–210 ppm)		
	Alkyl C	*N*-alkyl/methoxyl C	Carbohydrate C	di-O-alkyl C	Total	Aryl C	*O*- aryl C	Total	Carboxyl/amide C	Ketone C	Total
1989	11.1 abc	3.6 de	13.6 d	7.9 cdef	25.1 cde	27.4 def	7.8 cde	35.3 cdef	22.8 d	5.7 a	28.6 ab
1999											
OM	9.5 abcd	6.2 a	16.8 abc	8.9 a	31.9 a	24.9 g	9.1 b	34.0 ef	22.3 de	2.2 bc	24.6 cd
1/2OM	9.7 abcd	6.3 a	18.8 a	8.8 ab	33.9 a	25.5 fg	7.9 cd	33.4 f	20.2 e	2.9 abc	23.1 d
NPK	9.9 abcd	4.9 bc	17.5 ab	8.3 abcd	30.7 ab	25.4 fg	7.9 cd	33.3 f	23.1 cd	3.0 abc	26.1 abcd
NP	8.9 bcd	3.8 cde	13.6 d	7.1 gh	24.5 cde	29.1 bcd	8.5 bc	37.6 abc	25.6 ab	3.5 abc	29.1 a
PK	10.6 abc	4.7bcd	14.6 bcd	7.8 defg	27.1 cd	29.1 bcd	7.3 de	36.4 bcde	24.5 abcd	1.4 c	25.9 abcd
NK	11.9 a	3.7 de	12.8 d	6.8 h	23.2 e	31.4 ab	7.3 de	38.7 ab	22.9 cd	3.3 abc	26.2 abcd
CK	11.8 a	5.8 ab	14.3 cd	7.0 gh	27.1 cd	29.1 bcd	6.8 e	35.9 bcdef	23.2 bcd	2.0 bc	25.2 bcd
2009											
OM	7.8 d	4.7 bcd	14.6 cd	8.6 abc	27.8 bc	26.6 efg	10.6 a	37.2 bcd	24.8 abcd	2.5 bc	27.2 abc
1/2OM	8.7 cd	4.6 cd	14.5 cd	8.2 abcd	27.3 bcd	28.1 cde	9.4 b	37.4 abc	24.1 abcd	2.4 bc	26.5 abc
NPK	10.1 abcd	4.4 cd	15.4 bcd	7.2 fgh	27.0 cd	28.0 cde	7.6 cde	35.6 cdef	25.3 abc	2.1 bc	27.3 abc
NP	11.4 ab	4.9 bc	14.3 cd	8.0 bcde	27.2 cd	28.3 cde	7.7 cde	36.1 bcdef	22.6 de	2.8 abc	25.4 bcd
PK	11.3 abc	4.7 bcd	13.7 d	7.9 cde	26.4 cde	26.5 efg	7.8 cde	34.3 def	23.1 cd	4.9 ab	28.0 abc
NK	7.9 d	3.0 e	13.6 d	7.3 efgh	23.9 de	32.4 a	7.9 cd	40.3 a	25.9 a	2.0 bc	27.9 abc
CK	9.7 abc	3.9 cde	14.8 bcd	7.6 defgh	26.2 cde	30.4 abc	7.8 cde	38.2 abc	23.0 cd	2.9 abc	25.9 bcd

Treatments followed by different letters in a column are significantly different at *p* < 0.05 (Tukey test, n = 3).

The relative proportions of four major functional groups (alkyl C, O-alkyl C, aromatic C and carbonyl C) are generally similar in soils under the three balanced fertilization treatments (i.e. OM, 1/2OM and NPK) in both 1999 and 2009 (Table 1). Among the unbalanced fertilization treatments, the soils under NK treatment tended to contain the smallest fraction of O-alkyl C and the largest fraction of aromatic C in both years.

The proportion of alkyl C in soils did not seem to change with time of fertilizer application. The O-alkyl C in the balanced fertilization treatments increased by 22–36% during 1989–1999, and then decreased by 12–20% during 1999–2009, while the O-alkyl C in the unbalanced fertilization and CK treatments did not change with time of fertilizer application (Table 1). The relative abundances of aromatic C from the balanced fertilization treatments remained relatively stable during 1989–1999, and then increased by 7–13% during 1999–2009. The relative abundances of aromatic C from the unbalanced fertilization and CK treatments were generally unaffected by the time of fertilizer application, except for a significant increase of 4% during 1989–1999 under the NK treatment. The carbonyl C proportions from the OM and 1/2OM treatments decreased by 14% and 20%, respectively, during 1989–1999, but increased by 15% during 1999–2009. In other treatments, there were no changes for carbonyl C with time of fertilizer application. Comparisons among different fertilization treatments showed significantly larger proportions of O-alkyl C and smaller proportions of aromatic C in the balanced than in unbalanced fertilization and CK treatments in 1999, but no significant differences in 2009. Our results indicated that the distribution of C functional groups in soils fluctuated over the 20 years of continuous application of balanced fertilizers, but was relatively stable under unbalanced fertilization and CK treatments.

**Fig 3:**
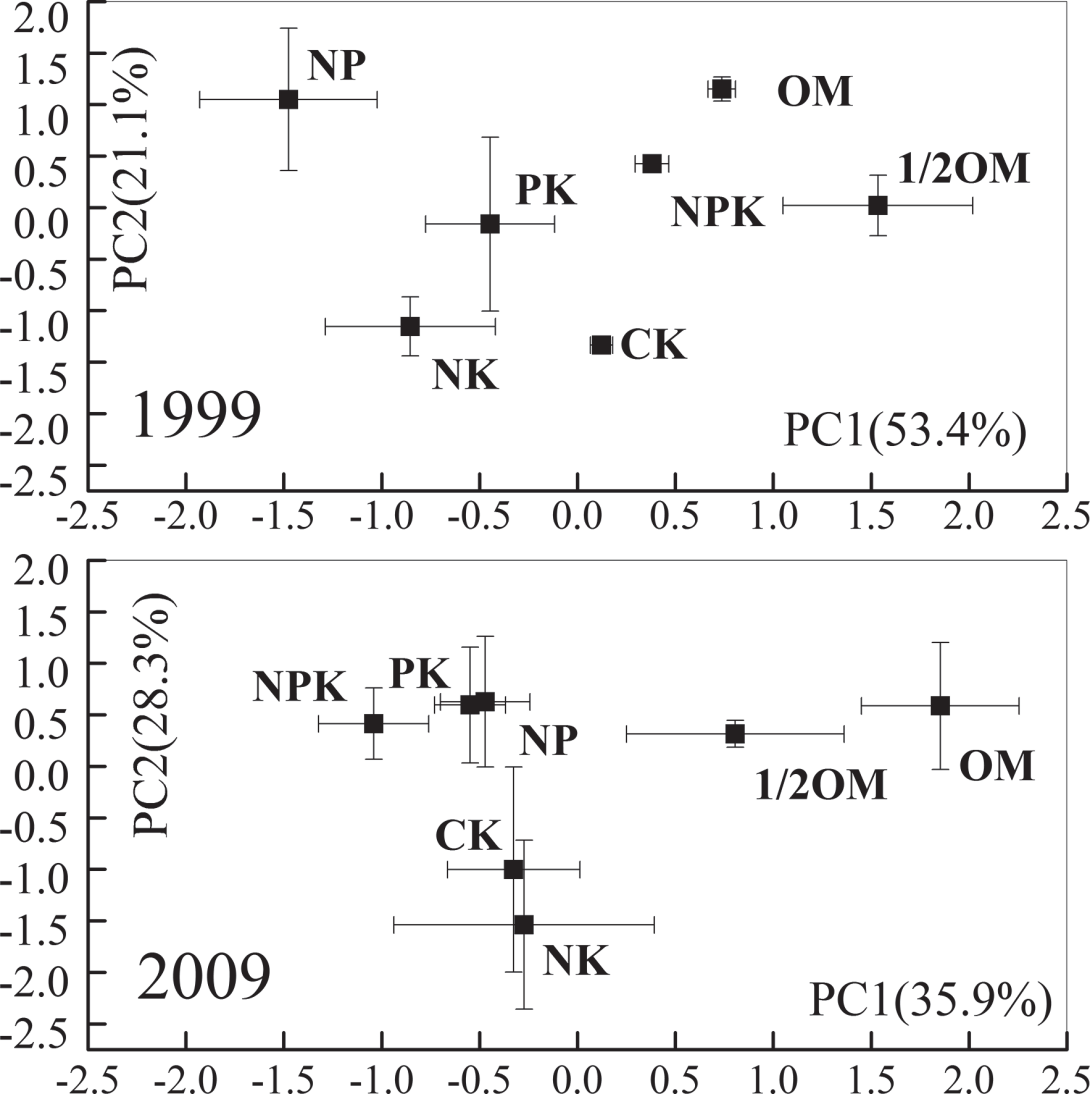
Principal component analysis of the composition of functional groups obtained from 1999 and 2009, separately. Vertical and horizontal bars represent the standard deviations (*n* = 3). Each point represents a specific SOC chemical structure in fertilization or CK treatments. Points that are close together are more similar to one another than points that are far apart.

Principal component analysis (PCA) based on the relative abundances of functional groups showed that SOC chemical composition responded significantly to the fertilization strategies, while the patterns differed between 1999 and 2009 (Fig 3). Specifically, the points reflecting fertilization treatments were more scattered in 1999 than in 2009, as indicated by the following two facts. First, the PC1 and PC2 values ranged from—1.48 to 1.53 and—1.34 to 1.15, respectively, in 1999, and in 2009 from—1.04 to 1.85 and—1.54 to 0.63, respectively; that is, PC1 and PC2 axes had wider ranges in 1999 than in 2009. Second, the points were significantly separated from each other in most cases in 1999, while in 2009 three clusters could be identified. In 2009, the OM and 1/2OM treatments were clearly separated from all others on PC1 and formed a first cluster. The NPK, NP and PK treatments formed a second cluster. The third cluster contained NK and CK treatments. These findings indicate that the functional groups from various fertilization strategies tended to become compositionally similar with time of fertilizer application.

## Discussion

### Change in SOC content

In this study, soils had significantly higher SOC content under the continuously balanced (i.e. OM, 1/2OM and NPK) than the unbalanced fertilization (i.e. NP, PK and NK) and CK treatments, and the differences were greater in 2009 than 1999 (Fig 1). The C inputs for the OM and 1/2OM treatments included direct application of organic compost and indirect introduction of root residues and exudates (the aboveground biomass was removed during experiments). For the other treatments, root residues and exudates were the only sources of organic C input into the soils.

Positive relationships between C inputs from the applied organic material and the resultant SOC content have been reported [27, 28]. Balanced fertilization resulted in higher crop yield than the unbalanced fertilization and CK treatments [12], indicating retention of greater amounts of root residues and exudates in soils in the former treatments. The SOC accumulation depends on the balance of C inputs and decomposition. Studies based on the same experimental field as used in the present study estimated that the average amount of exogenous organic C input in the OM, 1/2OM and NPK treatments during 1989–2009 was about 3.50–5.40 Mg C ha^−1^ yr^−1^ [29], while the annually mineralized organic C measured in 2002–2003 was about 3.46–4.01 Mg C ha^−1^ yr^−1^ [30], suggesting a net increase in soil C. In the PK, NK and CK treatments, however, the corresponding average amount of input C was about 0.93–1.35 Mg C ha^−1^ yr^−1^ [29], while mineralized organic C reached 1.99–2.19 Mg C ha^−1^ yr^−1^ [30], indicating a net decrease in soil C. Although a net increase in SOC (input 3.22 vs. output 3.13 Mg C ha^−1^ yr^−1^) in the NP treatment was also suggested based on the estimates by Fan et al. [29] and Ding et al. [30], the SOC content decreased during 1999–2009, likely due to the decreased C input, which is consistent with the decreased crop yield from long-term K deficiency [31].

### Change in SOC chemical structure

Balanced fertilization (i.e. OM, 1/2OM and NPK) treatments resulted in larger relative proportion of O-alkyl C but smaller aromatic C contents in 1999 than unbalanced fertilization and CK treatments (Table 1). The relative proportions of O-alkyl C decreased and those of aromatic C increased, however, during 1999–2009 in the balanced fertilization treatments. Changes in C functional group compositions in the unbalanced fertilization and CK treatments during 1989–2009 were not significant. PCA also showed that the distributions of C functional groups in soils from various fertilization strategies tended to become compositionally similar with time of fertilizer application.

In this study, the higher O-alkyl C proportion in balanced fertilization treatments in 1999 could be attributed to the introduction of organic compost and the higher root residues retained in soils (both rich in cellulose and lignin components), compared with unbalanced fertilization and CK treatments. The accumulation of microbially derived polysaccharides might also contribute [32], because it has been shown that the microbial biomass and activity were significantly higher in the balanced than unbalanced fertilization treatments [12]. The O-alkyl C is usually regarded as labile C for soil microbial communities, and has been shown to first lose signal intensity during organic C decomposition among all four main C functional groups [1]. Therefore, the accumulation of O-alkyl C components in the balanced fertilization treatments may suggest the presence of certain protection mechanisms of O-alkyl C components. Dungait et al. [33] has reported that labile compounds such as polysaccharides can persist for decades if they are not in contact with microbes. A previous study based on the same NCP experimental field demonstrated that balanced fertilization promoted the formation of macro-aggregates [34], which potentially allows for the physical isolation of O-alkyl C components located inside the aggregates. This isolation might reduce the possibility of microbial contact or attraction to O-alkyl C components.

The larger relative proportion of aromatic C in the unbalanced fertilization and CK treatments than the balanced fertilization treatments in 1999 may result from selective preservation of lignin structures during microbial decomposition, especially when some specific microorganisms (such as fungi) and oxidative enzymes capable of degrading lignin have a low biomass content and/or weak activity. Smaller fungi populations and weak activities of oxidative enzymes (i.e. dehydrogenase) have been noted in soils for unbalanced fertilization and CK than for balanced fertilization treatments in the same NCP experiment field [12, 14]. Gallo et al. [35] also reported that the decrease in oxidative enzyme activities is generally linked to the increased the proportion of aromatic C, as observed in northern United States hardwood forests. In addition, the smaller proportion of O-alkyl C (the main component of energy-rich labile C) associated with the unbalanced fertilization and CK treatments may further depress microbial growth and consequently slow the decomposition of recalcitrant organic C (such as lignin C), compared with the balanced fertilization treatments. Mahieu et al. [20] showed a negative correlation between the proportion of O-alkyl C with that of aromatic C in SOC, consistent with the results of the present study.

The observation that the composition of functional groups in soils under various fertilization strategies became similar with time of fertilizer application may be explained by homogenization of the organic matter during decomposition. However, this proposed homogenization in soils has not been investigated directly based on the evolution of SOC structure. Wang et al. [36] reported that the chemical structure of maize and wheat straw differed in the first half-year but became similar after two years of decomposition. Hsu and Lo [37] also pointed out that the composting process transformed heterogeneous raw pig manure to a compositionally uniform product. These represent time-dependent changes in chemical structure of organic matter.

In the present study, the SOC content continued to increase during 1989–1999 and 1999–2009 under balanced fertilization treatments (Fig 1). However, the relative proportion of O-alkyl C increased during 1989–1999, and then decreased during 1999–2009; relative aromatic C proportion remained constant during 1989–1999, and then increased during 1999–2009 (Fig 1 and Table 1). This indicates that the increase of the O-alkyl C proportion mainly contributed to the SOC increase during the first 10 years of balanced fertilization; during the second 10 years the increasing SOC could be due to accumulation of aromatic C components such as lignin. Such inconsistent changes in SOC and O-alkyl C proportion were observed in previous studies, in which labile C pools such as particulate organic C and light-fraction organic carbon C preferentially increased during the initial SOC accumulation process [6, 38]. These labile C pools all contain higher proportions of O-alkyl C than other C pools [23, 39].

In the unbalanced fertilization and CK treatments, the consistent decrease in SOC content during 1999–2009 was accompanied by non-significant changes of the relative proportions of O-alkyl C and aromatic C (Fig 1 and Table 1). This might suggest the decomposition of exogenous organic matter (i.e. roots and exudates), as well as stimulated decomposition of original SOC resulting from the annual inputs of new organic material. The relatively constant distributions of C functional groups during the latter 10 years observed here suggested no preferential decomposition of certain functional groups such as labile O-alkyl forms over the others under these treatments.

Among the three balanced fertilization strategies, we found no difference in the distribution of four major C types (Table 1), consistent with the study of Yan et al. [10] showing that the chemical structure of SOC was similar between treatments receiving pig manure and chemical fertilizers in paddy or upland soils. However, Wang et al. [6] and Zhang et al. [9] reported that addition of organic material resulted in higher O-alkyl C than addition of chemical fertilizers, and attributed this difference to the incorporation of increased carbohydrate and cellulose structures from exogenous organic material into SOC. The different results may also arise from different environmental conditions, tillage management and different types of organic material used.

The NK treatment generally resulted in the highest relative proportion of aromatic C and the lowest proportion of relative O-alkyl C in both 1999 and 2009 (Table 1), indicating that long-term P deficiency may increase the recalcitrance of SOC. Zhao et al. [12] found that the most limiting nutrient for plant growth was P in the NCP experiment field and also found the highest available N accumulation in the NK among all treatments. The highest relative proportion of aromatic C in the NK treatment may be attributed to (1) reduced production of lignin-degrading enzymes by white-rot fungi due to increasing available N levels [40], which may result in relative accumulation of aromatic C derived from the lignin, and (2) biochemical protection by binding N to the aromatic C rings, leading to the formation of more recalcitrant composites [41]. However, a significant loss of O-alkyl C with increasing N availability has also been reported [42].

Previous studies have shown that the SOC chemical structure may vary with SOC fractions [4, 43]. Cao et al. [4] reported that particulate organic matter fractions were dominated by carbohydrates, while humic acid fractions by aromatic C and COO/N-C = O groups. Additionally, Mao et al. [43] showed that calcium humate exhibited higher aromaticity, while mobile humic acid had lower aromaticity and greater contributions from lignin aromatic ethers using NMR spectral-editing techniques. It is very likely that chemical composition of various SOC fractions may respond differently to long-term fertilization strategies.

In the NCP, SOC content is a predominant indicator of soil fertility [12]. In this study, there were higher SOC contents in balanced than in unbalanced fertilization and CK treatments in 1999 (Fig 1). This may be attributed to the accumulation of O-alkyl C, as indicated by the higher proportion of O-alkyl C in balanced fertility (Table 1). Previous studies have shown that labile organic C fractions with a predominance of O-alkyl C could preferentially accumulate during the initial increasing process of SOC [6, 38]. The proportions of four major C types became similar by 2009 (Table 1), while SOC content continued to be higher in balanced than in unbalanced fertilization and CK treatments (Fig 1), leading to the absolutely higher contents of O-alkyl C and aromatic C in the former than the latter treatments.

## Conclusions

Balanced fertilization (i.e. OM, 1/2OM and NPK) treatments resulted in a significantly higher SOC content compared with unbalanced (i.e. NP, PK and NK) and CK treatments. The SOC content in the balanced fertilization treatments increased, while that in the unbalanced and CK treatments decreased with the time of fertilizer application. Unlike the trend in SOC content, the SOC chemical composition tended to become similar with time, as reflected by the fact that (1) significantly greater proportions of O-alkyl C and smaller proportions of aromatic C were found in the balanced than in the unbalanced fertilization and CK treatment after 10 years of fertilization, while the relative abundances of these two C functional groups in the former treatments become similar to those in the latter treatments after 20 years of fertilization, and (2) the points representing specific SOC chemical composition in various fertilization or CK treatments resulted from PCA were more scattered after 10 than after 20 years of fertilizer application.
